# Impaired Evidence Accumulation as a Transdiagnostic Vulnerability Factor in Psychopathology

**DOI:** 10.3389/fpsyt.2021.627179

**Published:** 2021-02-17

**Authors:** Chandra Sripada, Alexander Weigard

**Affiliations:** Department of Psychiatry, University of Michigan, Ann Arbor, MI, United States

**Keywords:** computational psychiatry, evidence accumulation, transdiagnostic, research domain criteria, schizophrenia, bipolar disorder, attention-deficit/hyperactivity disorder

## Abstract

There is substantial interest in identifying biobehavioral dimensions of individual variation that cut across heterogenous disorder categories, and computational models can play a major role in advancing this goal. In this report, we focused on efficiency of evidence accumulation (EEA), a computationally characterized variable derived from sequential sampling models of choice tasks. We created an EEA factor from three behavioral tasks in the UCLA Phenomics dataset (*n* = 272), which includes healthy participants (*n* = 130) as well-participants with schizophrenia (*n* = 50), bipolar disorder (*n* = 49), and attention-deficit/hyperactivity disorder (*n* = 43). We found that the EEA factor was significantly reduced in all three disorders, and that it correlated with an overall severity score for psychopathology as well as self-report measures of impulsivity. Although EEA was significantly correlated with general intelligence, it remained associated with psychopathology and symptom scales even after controlling for intelligence scores. Taken together, these findings suggest EEA is a promising computationally-characterized dimension of neurocognitive variation, with diminished EEA conferring transdiagnostic vulnerability to psychopathology.

## Introduction

The standard approach to psychiatric nosology, reflected in the widely used DSM (1) and ICD (2) systems, emphasizes discrete diagnostic categories, each assumed to have its own distinct pathophysiology. An alternative approach, central to the Research Domain Criteria (RDoC) initiative (3–5), understands mental disorders in terms of fundamental biobehavioral dimensions of variation that span disorders (6, 7). In this second approach, there is a critical need to identify these fundamental dimensions of variation, which are assumed to operate at a latent mechanistic level and may not have easily appreciated, one-to-one relationships with observable symptoms (3).

Computational psychiatry (8–12) is a research field that aims to formally model complex behaviors, typically performance during carefully constructed experimental tasks, in order to better understand abnormal patterns of functioning in psychiatric disorders. Standard models of task performance used in this field typically feature a small number of parameters that reflect latent psychological functions, and which might represent candidate biobehavioral dimensions on which individuals vary in clinically-significant ways. Here we focus on sequential sampling models (SSMs) (13–16), a class of models developed in mathematical psychology that are widely-used in cognitive neuroscience and have a substantial track record of success in explaining behavioral and neural phenomena on choice tasks (17–19).

**Figure 1:**
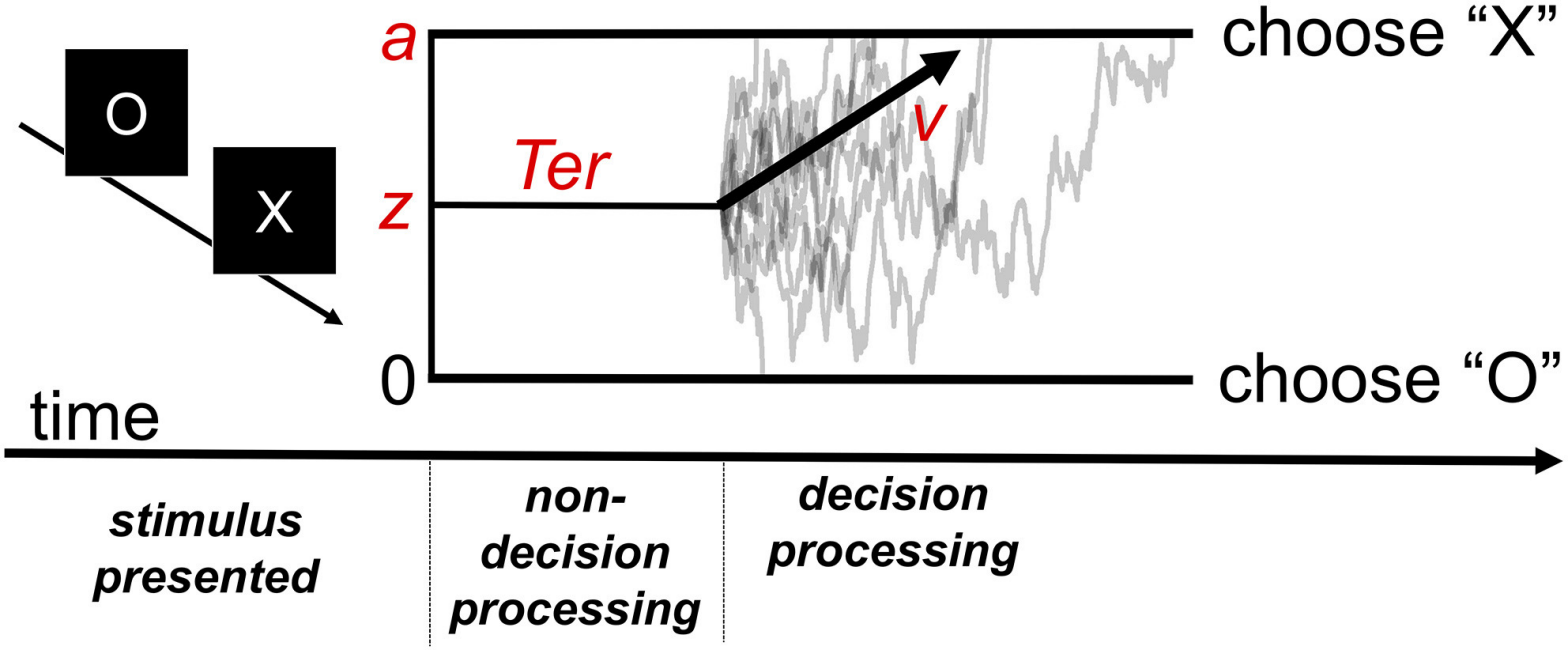
The diffusion decision model (DDM). The model explains performance in a simple decision task in which participants must decide whether the stimulus presented was an “X” or “O.” Following stimulus presentation, the decision process drifts between the boundaries for each possible response. Gray traces represent the paths of the stochastic decision processes for individual trials. The black arrow represents the critical drift rate parameter, **v**, which determines the average rate at which the process drifts toward the correct response boundary (e.g., toward an “X” response on a trial in which an “X” stimulus was presented). The **z** parameter represents the starting point bias of the process (in the example, it is set midway between the two decision boundaries representing absence of bias). The **a** parameter determines where the upper boundary is set (with the lower boundary always set at 0) and can be used to index “response caution,” the quantity of evidence that is required before the decision boundary is reached. The model also includes a parameter for time spent on processes peripheral to the decision (**Ter**).

SSMs aim to capture performance on forced choice tasks in which subjects select from two or more response options—a class of tasks that subsumes a large portion of experimental cognitive paradigms. In the diffusion decision model (DDM) (14, 15, 20), a popular and well-validated SSM, task performance is described by a noisy decision process that drifts over time toward one of two decision boundaries. The average rate at which the process moves toward the correct decision boundary is determined by the “drift rate” parameter *v*. The model also includes several additional parameters that determine other aspects of the decision process, which are discussed in Figure 1.

Although the DDM and related SSMs are used in experimental contexts to simulate and measure a wide variety of latent neurocognitive processes, the drift rate parameter is often of key interest. Individual differences in this parameter reflect the ability of the system to efficiently accumulate stimulus information that is relevant to selecting an appropriate response in the context of noisy information. Importantly, SSMs allow the drift rate to be disentangled from multiple other factors that affect task performance, which are indexed with the other model parameters, yielding much more precise estimates.

Trait efficiency of evidence accumulation (EEA), as indexed by the drift rate in SSMs, has several properties that make it attractive as a candidate transdiagnostic dimension. First, people exhibit stable individual differences in EEA (21, 22), suggesting it has trait-like qualities. Second, EEA manifests across a wide variety of tasks, and individual differences in EEA are correlated across these tasks (22, 23). This suggests EEA has some generality in influencing performance across multiple psychological domains. Third, EEA has been observed to be diminished in several psychiatric disorders (16, 24, 25), though most studies thus far have examined attention-deficit/hyperactivity disorder (ADHD) (26–29), and different tasks were used across these studies, making comparisons across disorders difficult. Fourth, recent work links EEA conceptually as well as empirically to poor self-regulation and impulsive decision-making (“impulsivity”) (30–32). Since impulsivity is itself found to be elevated across multiple psychiatric disorders (33, 34), a connection between EEA and impulsivity adds to the evidence that EEA might be implicated in psychopathology transdiagnostically.

The present study investigates EEA across multiple psychiatric disorders, while addressing some of the limitations in previous research. We take advantage of the publicly-available UCLA Phenomics dataset, a large sample (*n* = 272, with 142 having schizophrenia, bipolar disorder, or ADHD) that was extensively characterized with structured clinical interviews, self-report scale measures of symptom dimensions, behavioral measures of cognition, and a number of forced choice tasks that can be analyzed in an SSM framework (35). We constructed an EEA factor from three behavioral tasks available in this sample, the Stroop task, Go/No Go task, and the Stop Signal task. We hypothesized that EEA would be significantly reduced across three disorder categories, and that it would be significantly negatively associated with global psychopathology severity and trait impulsivity.

## Methods

### Participants

Participants were recruited as part of a larger study within the Consortium for Neuropsychiatric Phenomics at University of California, Los Angeles (www.phenomics.ucla.edu). Healthy controls were recruited through community advertisements from the Los Angelos area, while patients were recruited through outreach to local clinics and online portals. All participants were between ages 21 and 50, had no major medical illnesses, and provided urinalyses negative for drugs. Healthy controls in addition were excluded if they had a lifetime diagnosis of schizophrenia, bipolar disorder or substance abuse/dependence, or current diagnosis of other psychiatric disorders. All participants underwent structured clinical interviews, neuropsychological testing, and administration of a number of behavioral tasks. They in addition underwent two fMRI scanning sessions, but imaging data is not part of the present analysis. Comprehensive descriptions of the sample and measures collected are available elsewhere (35). Study procedures were approved by the UCLA institutional review board and all participants provided written informed consent.

The present analysis relies on a sample of 272 subjects. Psychiatric diagnosis was established by the Structured Clinical Interview for DSM-IV (36). Primary diagnoses and demographic information are shown in Table 1. As shown in this table, participants with schizophrenia and participants with bipolar disorder were younger than healthy participants. In addition, participants with schizophrenia were more likely to be male and had fewer years of school. Thus, we report results with statistical correction for these demographic factors. Some subjects were missing data for certain measures (Stroop Task *n* = 2, Stop Signal Task *n* = 2). As a result, the sample size available for most comparisons was 268 subjects.

**Table 1:** Demographic characteristics of the UCLA Phenomics sample.

	**Healthy**	**Schizophrenia**	**Bipolar I**	**ADHD**
n	130	50	49	43
Age (mean, sd)	31.3 ± 8.7	36.5 ± 8.9^***^	35.3 ± 9.0^**^	33.1 ± 10.8
Gender (#male)	68	38^**^	28	21
Years schooling	15.0 ± 1.7	12.6 ± 1.7^***^	14.6 ± 2.0	14.6 ± 1.8

Statistically different than healthy controls at:

*** *p < 0.001*,

** *p < 0.01*.

### Behavioral Tasks and Construction of an Evidence Accumulation Factor

Three behavioral tasks in the UCLA Phenomics dataset met the basic assumptions of SSMs. In particular, they were all choice tasks with discrete response alternatives, all afforded relatively rapid responses (under 1.5 s mean reaction time), and all had sufficient number of trials (ranging from 54 to 360 trials per task/condition). Full descriptions of all measures are available at the UCLA Phenomics Wiki page (http://lcni-3.uoregon.edu/phenowiki/index.php/HTAC). Brief descriptions follow:

***Color/Word Stroop Task***–This is a computerized version of the traditional Stroop task in which individuals are asked to respond with the ink color (red, green or blue) of a word stimulus. The meaning of the word is either congruent with the color (e.g., “RED” in red coloring) or incongruent, matching a different possible color response (e.g., “BLUE” in red coloring). Participants were presented with 98 congruent and 54 incongruent trials in pseudorandom order. Each stimulus was presented for 150 ms following a 250 ms fixation cross and participants were allowed to respond during a subsequent 2,000 ms interval. Responses were immediately followed by an 1,850–1,950 ms blank screen delay.

***Go/No-Go Task***–Participants were presented with a sequence of letters and were instructed to press a button as quickly as possible after the presentation of any letter except “X,” but to withhold their response after the presentation of “X.” The task contained 18 blocks presented in random order, with six blocks in each of three inter-trial interval (ITI) conditions: 1,000, 2,000, and 4,000 ms. Each stimulus was presented for 250 ms and followed by a blank screen response interval of 750, 1,750, or 3,750 ms depending on the ITI condition. Each block contained 20 trials (two of which were “X” trials) for a total of 360 trials across the entire task.

***Stop Signal Task***–Participants were presented with a series of “X” and “O” stimuli for 1,000 ms and were asked to press a button corresponding to the stimulus presented on that trial. Trials were preceded by a 500 ms fixation cross and followed by a 100 ms blank screen interval. On a subset of trials (25%, “stop” trials) an auditory tone which indicated that the participant should withhold their response to that trial (“stop signal”) was played following the stimulus presentation. The latency of the stop signal following the stimulus onset, or “stop signal delay” (SSD), was determined using a standard staircase tracking algorithm which dynamically adjusted the SSD on each “stop” trial in 50 ms increments with the goal of obtaining an inhibition rate of ~50% for each individual. Participants completed two blocks of 32 “stop” trials and 96 “go” (i.e., non-“stop”) trials each. The task's primary dependent measure of response inhibition, stop-signal reaction time (SSRT), which is thought to index the latency of a top-down process that inhibits responses on “stop” trials (37), was estimated using a quantile-based method outlined by (38). For the model-based analysis, we focused exclusively on the 192 “go” trials, following previous applications of SSMs to the stop signal task (26, 30, 39).

The UCLA Phenomics dataset does not provide trial-level data for these tasks, but instead provides detailed per-subject summary statistics characterizing a wide variety of task dimensions. Thus, we used the “EZ-diffusion model” (EZDM) approach (40). This method provides a closed-form analytic solution for estimating the main parameters of the DDM (see Introduction). Inputs to the EZDM procedure are three subject-level summary statistics: the proportion of correct decisions, the mean of correct response times, and the variance of correct response times. The EZDM procedure produces parameter estimates for individuals' drift rate (*v*), boundary separation (*a*: an index of response caution), and non-decision time (*Ter*: time taken up by perceptual and motor processes peripheral to processing of the choice).

EZ-diffusion model has been shown to produce parameter estimates and inferences that are highly similar to those drawn from more complex modeling methods (41, 42), and some data suggests it recovers individual-differences—the main interest in this study—better than model-fitting methods that use trial-to-trial data (43). Previous comprehensive simulation studies suggest that EZDM can precisely recover drift rate with roughly 70 trials per task (44). Since the trial counts (i.e., 98, 54,192, and 360) for our task conditions typically exceed this number, we can be reasonably confident that drift rates for these tasks were accurately recovered, and even more confident that the latent factor, which draws strength across trials in all four conditions, was accurately recovered. We also note that, although the DDM is intended to describe two-choice tasks, it is possible to obtain comparable parameter estimates for three-choice paradigms, such as the current study's Stroop task, under the assumption that correct and error responses are similar across trials requiring different choices (15). Hence, we adopted this simplifying assumption in order to allow drift rate estimates to be obtained from the Stroop task in this unique data set.

EZ-diffusion model was applied separately to four task conditions: Stroop congruent trials, Stroop incongruent trials, Go/No-Go trials, and Stop Signal “go” trials. Parameters were estimated in R (45) using the code from the original EZDM manuscript (40) with the scaling parameter (within-trial drift variability) set at 1. For the Go/No-Go task, proportion correct was computed by considering all trials (i.e., both “X” and non-“X”), and the response time mean and variance was set to the values for correct hits (trials on which individuals correctly inhibited their response do not have an observed response time), a method similar to that used in (27). For participants with perfect accuracy on any task, the edge correction recommended in the original article was used: We replaced the proportion correct value of 1 with a value that corresponds to the output of the following equation, where *n* is the number of trials in the task:

$$\begin{array}{l} 1-\frac{1}{2n} \end{array}$$

**Figure 2:**
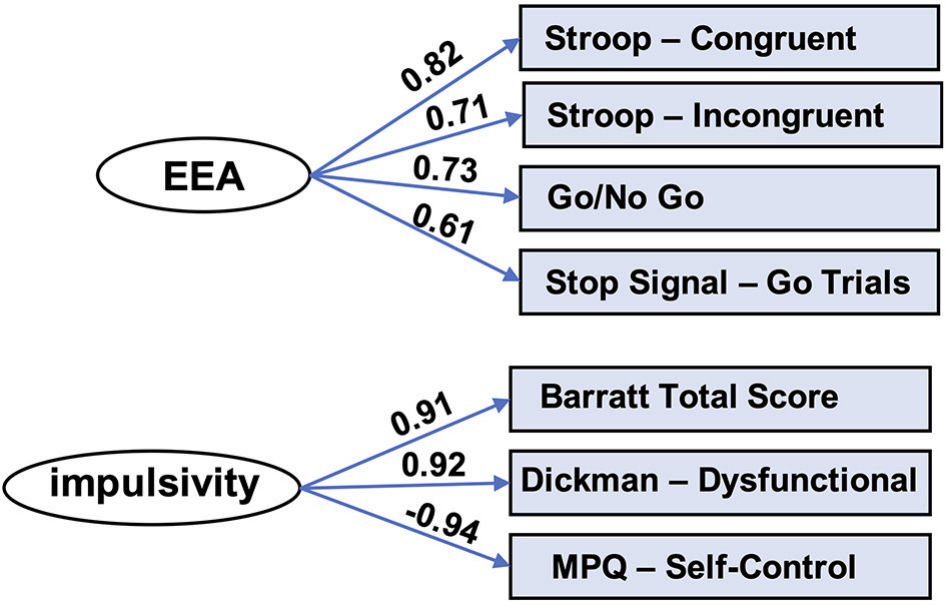
Factor models for EEA and impulsivity. Factor analysis yielded latent variables for efficiency of evidence accumulation (EEA) and impulsivity. MPQ, Multidimensional Personality Questionnaire.

Next, factor analysis, implemented in SPSS 25 (IBM, Armonk, NY), was used to produce an EEA factor from the drift rate parameters derived from the four task conditions. This factor accounted for 50% variance of the variance in the drift rates. Factor loadings were, respectively, for the four task conditions listed above: 0.82, 0.71, 0.73, and 0.61. Cronbach's alpha was 0.69, and it did not increase by dropping any items (Figure 2, top panel).

### Additional Neuropsychological and Symptom/Trait Factors

#### General Intelligence Factor

We produced a general intelligence factor from scores from nine cognitive tasks: WAIS-letter number sequencing, WAIS-vocabulary, WAIS-matrix reasoning, color trails test, WMS IV-symbol span, WMS IV-digit span, WMS IV-visual reproduction 1, WMS IV-visual reproduction 2, and California Verbal Learning Test. We used bifactor modeling, given prior results supporting the superiority of this type of model in this domain (46). In particular, we utilized code from Dubois and colleagues (47), which in turn uses the omega function in the psych (v 1.8.4) package (48) in R (v3.4.4) (45). The code performs maximum likelihood-estimated exploratory factor analysis (specifying a bifactor model), oblimin factor rotation, followed by a Schmid-Leiman transformation (49), yielding a general factor (“g”) as well as specific factors.

**Figure 3:**
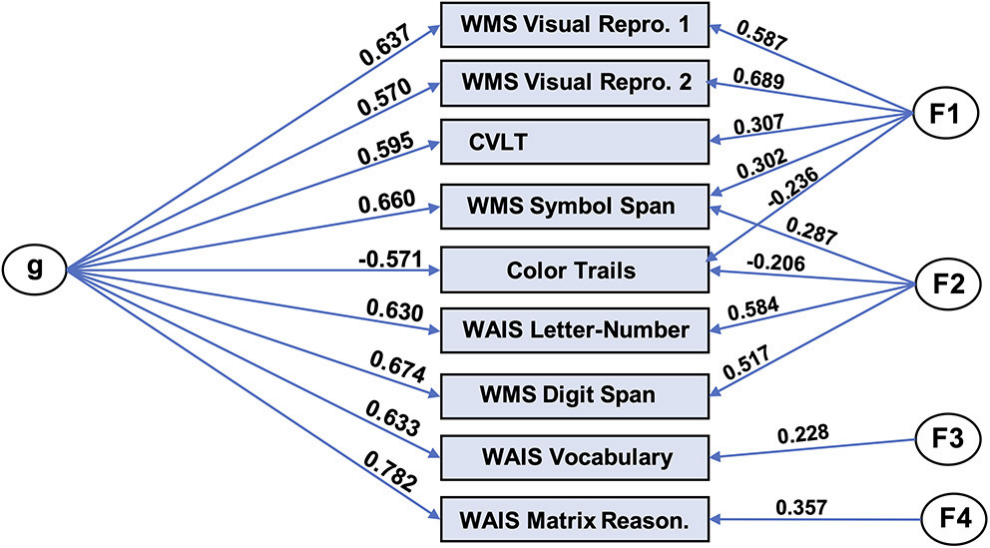
Bifactor model of general intelligence. Bifactor modeling was performed on nine cognitive tasks in the UCLA dataset. The resulting model consisted of a general factor (“g”) and four group factors and exhibited excellent fit with the data. WMS, Wechsler Memory Scale; Repro, Reproduction; WAIS, Wechsler Adult Intelligence Scale; Reason, Reasoning.

Using this code, we found a bifactor model fit the data very well by conventional standards (CFI = 0.991; RMSEA = 0.030; SRMR = 0.019; BIC = 7.23). The solution, which included a general factor and four group factors, is depicted in Figure 3. The general factor accounts for 72.2% of the variance [coefficient omega hierarchical ω (50)], while the four specific factors accounted for 16.7% percent of the variance.

#### Trait Impulsivity Factor

We used factor analysis to produce a trait impulsivity factor from scores on three self-report scales measuring impulsivity: Barratt Impulsivity Scale (total score) (51, 52), Dickman Impulsivity Scale (total dysfunctional) (53), and Multidimensional Personality Questionnaire (self-control subscale) (54). This factor accounted for 85% variance of the variance in the scores. Factor loadings for these scales, respectively, were: 0.91, 0.92, and −0.94. Cronbach's alpha was 0.77 and did not improve appreciably by removal of any of the scales (Figure 2, bottom panel).

#### General Severity Score for Psychopathology

Participants completed the Hopkins Symptom Checklist (55), a 56-item validated scale for symptoms associated with a broad range of psychiatric disorders. We used the global severity of psychopathology score from the Hopkins checklist, which is a summary score of total symptom load across all scale items.

## Results

### Evidence Accumulation Is Reduced Across Three Mental Disorders

**Figure 4:**
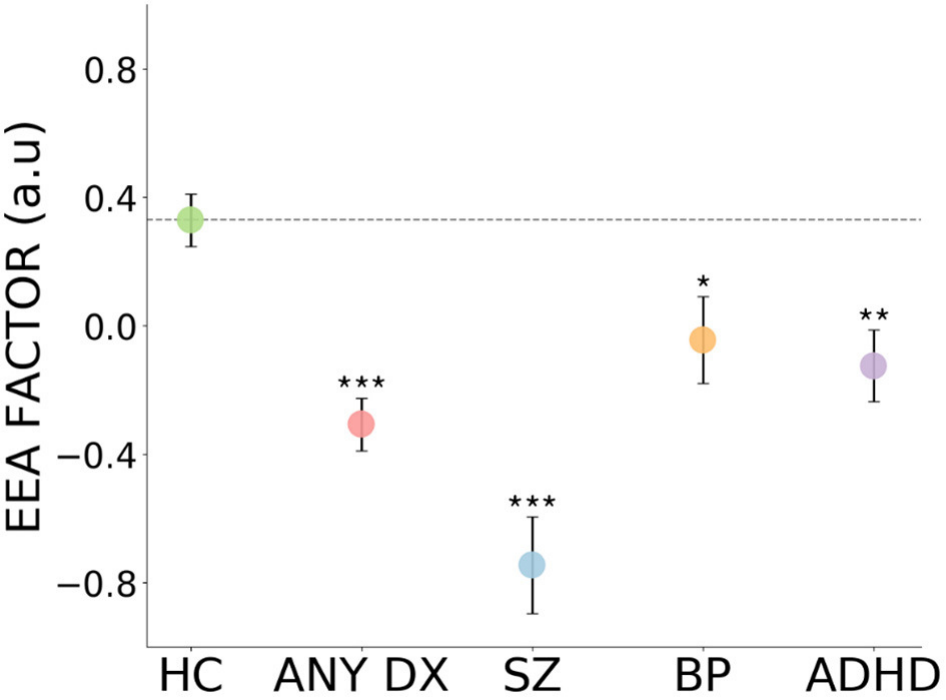
Impaired evidence accumulation across psychiatric diagnoses. The evidence accumulation factor is significantly reduced in all patients, collapsing across the three diagnoses, in comparison to healthy controls. It is also reduced in each patient groups separately in comparison to healthy controls. HC, Healthy Controls; ALL DX, All three patient groups combined; SZ, Schizophrenia; BP, Bipolar Disorder; ADHD, Attention-deficit/hyperactivity Disorder; a.u., arbitrary units. Error bars reflect represent standard error. ***p < 0.001, **p < 0.01, *p < 0.05.

As shown in Figure 4, Table 2, the EEA factor was significantly reduced in all patients (collapsing across the three diagnoses) in comparison to healthy controls. It was also significantly reduced in each patient group separately in comparison to healthy controls. Effect sizes ranged from large (schizophrenia) to medium (all diagnoses and ADHD) to small-to-medium (bipolar disorder). We repeated these analyses controlling for age, gender, and years schooling and differences remained highly statistically significant. Mean and standard deviations per group for all task summary metrics and clinical score variables are shown in Supplementary Table 1.

**Table 2:** Reduced efficiency of evidence accumulation (EEA) in healthy controls vs. patients with psychiatric disorders.

		**Comparison w/healthy controls**				**w/demographic controls**		
	**EEA mean (sd)**	***t***	**df**	***p*-value**	**Cohen's d**	**F**	**df**	***p*-value**
HC	0.3 (0.9)							
ALL DX	−0.3 (0.9)	5.48	266	<0.001	0.67	30.8	1,263	<0.001
SZ	−0.7 (1.0)	6.57	174	<0.001	1.12	43.5	1,171	<0.001
BP	−0.04 (0.9)	2.38	176	0.02	0.40	5.9	1,173	0.02
ADHD	−0.1 (0.7)	2.91	170	0.004	0.51	8.9	1,167	0.003

*EEA was significantly reduced across all disorder categories. HC, Healthy Controls; ALL DX, All three patient groups combined; SZ, Schizophrenia; BP, Bipolar Disorder; ADHD, Attention-deficit/hyperactivity Disorder. Demographic control variables are age, gender, years schooling*.

### Evidence Accumulation Is Inversely Associated With the Global Psychopathology Severity Score and Self-Reported Impulsivity

**Figure 5:**
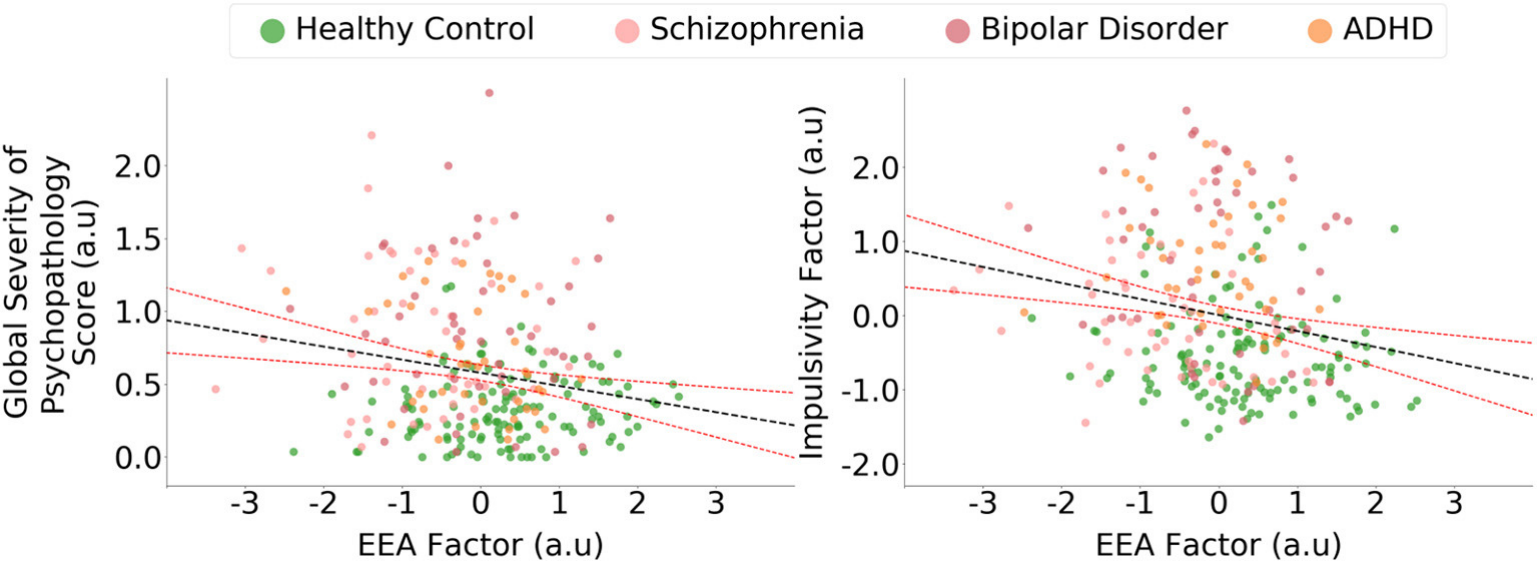
Evidence accumulation is related to global psychopathology and impulsivity. The efficiency of evidence accumulation (EEA) factor was statistically significantly correlated with a global psychopathology severity score derived from a self-report general symptom scale. The evidence accumulation factor was also significantly correlated with an impulsivity factor derived from three self-report scales of trait impulsivity. Confidence intervals represent 95% predictive intervals, a.u., arbitrary units.

Across the whole sample, the EEA factor was inversely correlated with the global psychopathology severity score (*r* = −0.20, *p* = 0.001; Figure 5), and this relationship remained significant after controlling for age, gender, and years of schooling [*t*_(263)_ = 2.6, *p* = 0.009]. The EEA factor was also inversely correlated with the impulsivity factor (r = −0.22, *p* < 0.001; Figure 5), and this relationship remained significant after controlling for age, gender, and years of schooling [*t*_(263)_ = 3.3, *p* = 0.001]. We also performed analyses that, in addition to demographic variables, include categorical regressors that control for the effect of individual psychiatric diagnoses (i.e., schizophrenia, bipolar, and ADHD). The relationship between EEA and impulsivity remained statistically significant [*t*_(259)_ = 2.0, *p* = 0.04], but the relationship between EEA and global psychopathology was not [*t*_(259)_ = 0.65, *p* = 0.52].

### Evidence Accumulation Remains Associated With Psychopathology After Controlling for General Intelligence

The EEA factor was correlated with general intelligence (*r* = 0.43, *p* < 0.001), consistent with EEA playing a general role in diverse forms of cognitive processing. Given this correlation, we assessed whether after controlling for general intelligence, the EEA factor remained related to psychopathology, the global psychopathology severity score, and impulsivity. We found that even after these controls, EEA remained statistically significantly reduced in all patients (collapsing across the three diagnoses) in comparison to healthy controls [*F*_(1, 265)_ = 34.4, *p* < 0.001, eta^2^ = 0.10]. It was also reduced in schizophrenia [*F*_(1, 173)_ = 47.7, *p* < 0.001, eta^2^ = 0.20], bipolar disorder [*F*_(1, 175)_ = 6.5, *p* = 0.01, eta^2^ = 0.03], and ADHD [*F*_(1, 171)_ = 9.8, *p* = 0.002, eta^2^ = 0.05]. Additionally, the EEA factor remained statically significantly associated with the impulsivity factor (standardized beta = −0.17, *p* = 0.003) and the global psychopathology severity score (standardized beta = −0.12, *p* = 0.03). We additionally examined all the preceding relationships with demographic controls (age, gender, years schooling), and found they all remained with levels of statistical significance that were essentially unchanged.

Additionally, we assessed the preceding relationships in healthy controls alone. In healthy controls, EEA remained significantly correlated with general intelligence (*r* = 0.40, *p* < 0.001), but not global psychopathology (*r* = −0.10, *p* = 0.22) and impulsivity (*r* = −0.08, *p* = 0.31).

### Evidence Accumulation Is More Reliably Linked to Psychopathology Than Traditional Dependent Measures From the Three Behavioral Tasks

We additionally assessed the relationship between more traditional dependent measures from the three behavioral tasks and measures of psychopathology. Table 3 shows five traditional dependent measures for these tasks (further clarification of these measures is found in the Table caption). We found that across 20 total comparisons, just five were statistically significant. It is also notable that in all five cases, the corresponding relationship between EEA and the respective measure of psychopathology was larger (compare Cohen's d values from Tables 2, 3).

**Table 3:** Relationship between standard dependent measures in three behavioral tasks and measures of psychopathology.

	**Stroop reaction time effect**	**Stroop accuracy effect**	**Go/No-Go false alarms**	**Go/No-Go d prime**	**Stop signal SSRT**
HC vs. ALL DX	*d = 0.12; p* = 0.30	*d = 0.17; p* = 0.16	***d=0.12; p*** **=** **0.30**	***d=0.33; p*** **=** **0.008**	***d=0.43; p*** **=** **0.001**
HC vs. SZ	*d = 0.06; p* = 0.70	***d=0.44; p*** **=** **0.009**	*d = 0.14*; ***p*** **=** **0.39**	***d=0.51; p*** **=** **0.001**	***d=0.91; p*** **<** **0.001**
HC vs. BP	*d = 0.07; p* = 0.66	*d = 0.11; p* = 0.49	*d = −0.04; p* = 0.81	*d = 0.23; p* = 0.14	*d = 0.21; p* = 0.20
HC vs. ADHD	*d = 0.23; p* = 0.18	*d = 0.17; p* = 0.33	*d = 0.21; p* = 0.24	*d = 0.23; p* = 0.17	*d = 0.17; p* = 0.32

*We computed Cohen's d for five standard dependent measures. Positive d (observed in 19 out of 20 cases) refers to better performance in healthy controls (HCs) compared to patients (specifically: smaller Stroop reaction time and accuracy effect, fewer Go/No-Go false alarms and higher d prime, and smaller Stop Signal SSRT. Of 20 comparisons, just five were statistically significant (highlighted in bold). Stroop Reaction Time Effect, difference in reaction time between the incongruent and congruent condition; Stroop Accuracy Effect, difference in accuracy between the incongruent and congruent condition; d prime, signal detection sensitivity parameter; SSRT, stop signal reaction time, i.e., the latency in initiating a stopping process after the presentation of the stop signal (this latency is inferred based on a model of task performance). ALL DX, All three patient groups combined; SZ, Schizophrenia; BP, Bipolar Disorder; ADHD, Attention-deficit/hyperactivity Disorder*.

## Discussion

Computational psychiatry opens up opportunities to rigorously identify and characterize clinically-significant biobehavioral dimensions of individual variation using precisely-specified mathematical models (8–12). This study appears to be the first to examine in a single group of subjects the relationships between evidence accumulation (EEA), a key computational parameter in sequential sampling models (SSMs) of choice tasks, and multiple measures of psychopathology diagnosis and symptoms. Our major findings are that: (1) EEA is significantly reduced in schizophrenia, bipolar disorder, and ADHD; (2) It is significantly negatively correlated with a global severity of psychopathology score as well as self-reported impulsivity; (3) EEA outperforms traditional metrics from behavioral tasks, such as reaction time and accuracy difference scores, in being more strongly associated with psychopathology and symptom scales. Taken together, these findings suggest diminished EEA is a promising computationally characterized transdiagnostic vulnerability factor in psychopathology.

Sequential sampling models are among the most widely used computational models in psychology and neuroscience (13–16). Key advantages of these models are that they capture complex and subtle features of response profiles across a broad range of psychological tasks (56). Previous studies have also found that EEA values, as indexed by drift rate parameters in SSMs, are related across tasks (21, 22) and stable across sessions (22, 23), suggesting they have trait-like properties. The current study extends these results by showing that EEA has broad, transdiagnostic relationships with psychopathology: It is reduced across all three disorder categories present in this sample, and it is inversely correlated with clinically relevant symptom scales.

Interestingly, while the EEA construct has been extensively invoked to explain task performance in hundreds of studies, the mechanistic basis of individual-differences in EEA, and how these differences relate to differences in other cognitive constructs, are much less explored; but see (39, 57, 58). Several studies using perceptual decision tasks find drift rates are related to general intelligence (59–62), similar to the pattern we found in the present study. But, again, similar to prior work, the correlations we found in this study between EEA and general intelligence were moderate in size, with individuals' general intelligence explaining <25% of the variance in EEA in this sample. Furthermore, we found that EEA continued to be related to multiple measures of psychopathology, including schizophrenia, ADHD, trait impulsivity, and global psychopathology severity, even after adjusting for individuals' general intelligence. Hence, although additional research is needed, current evidence suggests that EEA and intelligence are related, but psychometrically dissociable, individual-difference dimensions.

In contrast to EEA, standard dependent measures from the Stroop, Go/No-Go, and Stop Signal tasks performed relatively poorly in terms of being associated with psychopathology measures, with just five significant effects observed in 20 comparisons (without applying multiple comparisons correction), and these effects were scattered across different comparisons (see also (63, 64). We propose two explanations for why EEA was more strongly related to psychopathology than traditional metrics.

First, a recent pair of influential studies, each examining multiple behavioral tasks, found that standard dependent measures from these tasks exhibit relatively poor test-retest reliability (65, 66). These observations are further supported by theoretical work with simulations that shows that difference score metrics (for example, Stroop interference effects) fare particularly poorly (67, 68). One reason is that subtracting scores from one condition against another will, given plausible assumptions, increase noise relative to signal, thus diminishing test-retest reliability (67). Since the reliability of a measure sets a ceiling on how well it can correlate with another variable (69), the low reliability of standard metrics derived from these tasks could help to explain their weaker relationship with psychopathology. In contrast, EEA estimates from tasks with sufficient numbers of trials have generally been demonstrated to exhibit good reliability (e.g., *rs* > 0.70) (21), thus potentially enabling higher correlation with psychopathology.

A second possible interpretation for the lack of predictivity of standard metrics, one that is not mutually exclusive with the first, is that these metrics reflect complex interactions among multiple factors, some of which relate to psychopathology but others of which do not (67). Computational modeling can play a key role in disentangling these interacting factors, allowing researchers to identify underlying parameters that are more directly associated with clinically-significant dimensions (24–26, 70).

For example, standard dependent measures from behavioral tasks, such as task accuracy or reaction time, cannot distinguish whether lower values in an individual are due to lower intrinsic ability on the task or preferences to trade off lower accuracy for greater speed (or lower speed for greater accuracy) (71). Sequential sampling models, in contrast, provide an explicit model of this tradeoff (14, 56, 70), which allows the rate of evidence accumulation for the correct response option to be distinguished from the threshold of evidence at which a response is selected (where higher evidence thresholds correspond to greater preference for accuracy over speed). The results of this study provide support for the view that computational models enable better quantification of underlying dimensions of inter-individual variation and yield stronger relationships with measures of psychopathology (16, 25–27).

We found that EEA is reduced transdiagnostically, and the nature of EEA may shed light on why it manifests across diverse disorder types. Evidence accumulation is conceptualized as a basic ability to rapidly extract information from a stimulus to select contextually appropriate responses. This is a highly general ability and thus reductions in EEA would be expected to increase the probability that inappropriate responses are produced across diverse psychological domains (attention, threat detection, and motivation). Consistent with recent hierarchical models of psychopathology, which posit abnormalities at multiple levels of generality, other abnormalities might operate more locally in individual psychological domains (e.g., enhanced sensitivity of threat detection systems). Together, interactions between high-level abnormalities such as impaired EEA and more local domain-specific abnormalities could produce the actually observed clinical pattern involving both substantial covariance as well as specificity in psychiatric symptoms.

The current study has several limitations. First, we used an EEA factor derived from three tasks in the UCLA Phenomics sample that met basic assumptions of SSMs. Future studies should extend this work by examining a more diverse range of tasks. Second, given the availability of subject-level summary statistics, we used the EZDM approach for calculating SSM parameters, rather than performing trial-by-trial modeling. EZ-diffusion model results, however, usually correlate highly with results from trial-by-trial approaches, and some studies suggest EZDM can be more effective in quantifying individual differences (41–43). We also note that newer specialized modeling approaches are emerging for modeling “conflict” (e.g., Stroop) tasks in which unique features of these tasks are explained by a process in which correct responding requires overriding a pre-potent response (72–74). This is a fast-evolving area of research and future studies could employ these newer approaches. Relatedly, it is also possible that performance in the go/no-go task analyzed in this paper is affected by processes that are not described well by the simple EZDM model, e.g., response bias toward the upper boundary (75). However, we note that for tasks that similarly encourage response biases, such as the continuous performance task (CPT), initial findings of EEA deficits in ADHD from simple EZDM fits (27) have been strongly upheld after more comprehensive modeling (76). Finally, this sample included three major mental disorders, schizophrenia, bipolar disorder, and ADHD. A number of others disorders—for example, major depression, obsessive compulsive disorder, autism—were not well-represented in this sample, and future studies should examine a wider range of clinical populations to assess the robustness of our results across disorder categories.

In sum, this study demonstrates that EEA holds promise as a basic dimension of inter-individual neurocognitive variation, and impaired EEA may be a transdiagnostic vulnerability factor in psychopathology.

## Data Availability Statement

Code and links to the primary data are available at the following Open Science Framework (OSF) page: https://osf.io/cqksz/.

## Ethics Statement

The studies involving human participants were reviewed and approved by UCLA Institutional Review Board. The patients/participants provided their written informed consent to participate in this study.

## Author Contributions

CS and AW contributed to all aspects of the manuscript including study conception, data analysis, interpretation of results, and manuscript writing. Both authors contributed to the article and approved the submitted version.

## Conflict of Interest

The authors declare that the research was conducted in the absence of any commercial or financial relationships that could be construed as a potential conflict of interest.
