# Supplementary material for: Impaired Evidence Accumulation as a Transdiagnostic Vulnerability Factor in Psychopathology
Source: Front Psychiatry. 2021 Feb 17;12:627179. doi: 10.3389/fpsyt.2021.627179 (PMC7925621; doi:10.3389/fpsyt.2021.627179)
Supplement: Supplementary file 1 [file Table_1.DOCX]

**Supplemental Results**

|  | Stroop Reaction Time Effect | Stroop Accuracy Effect | Go/No-Go False Alarms | Go/No-Go  d prime | Stop Signal  SSRT | Impuls-ivity | Global Psycho-pathology | General Intell. |
| --- | --- | --- | --- | --- | --- | --- | --- | --- |
| HC | 125.1  (71.7) | -0.03  (0.05) | 12.7  (6.7) | 3.0  (0.7) | 224.3  (42.6) | -0.5  (.66) | 0.3  (0.2) | 0.3  (0.7) |
| SZ | 129.7  (71.1) | -0.05  (0.07) | 13.7  (6.8) | 2.7  (0.9) | 264.1  (46.4) | .03  (.87) | 0.8  (0.5) | -1.0  (0.8) |
| BP | 130.1  (54.3) | -0.03  (0.05) | 13.0  (7.9) | 2.9  (1.0) | 233.9  (50.4) | 0.8  (1.2) | 0.9  (0.5) | 0.0  (0.8) |
| ADHD | 142.5  (78.9) | -0.04  (0.06) | 14.2  (7.9) | 2.9  (0.9) | 231.4  (36.4) | 0.7  (0.8) | 0.7  (0.4) | 0.2  (0.7) |

***Table S1. Summary Statistics by Group for Tasks and Psychometric Variables****.* Entries are mean and standard deviation (in parentheses).
